# A CT-based radiomics nomogram involving the cystic fluid area for differentiating appendiceal mucinous neoplasms from appendicitis with intraluminal fluid

**DOI:** 10.1007/s00432-024-05695-5

**Published:** 2024-03-20

**Authors:** Xinbin Wang, Na Feng, Yonggang Qiu, Hao Dong, Cuncheng Lou, Junjie Yang, Jieni Yu, Chunyan Jiang, Jianxia Xu, Risheng Yu

**Affiliations:** 1 Department of Radiology, The Second Affiliated Hospital, Zhejiang University School of Medicine, 88 Jie-Fang Road, Hangzhou, 310009 Zhejiang China; 2 Department of Radiology, The First People’s Hospital of Xiaoshan District, Xiaoshan Affiliated Hospital of Wenzhou Medical University, Hangzhou, Zhejiang China; 3 Department of Pathology, The First People’s Hospital of Xiaoshan District, Xiaoshan Affiliated Hospital of Wenzhou Medical University, Hangzhou, Zhejiang China; 4 Department of Radiology, People’s Hospital of Songyang County, Lishui, Zhejiang China; 5 Department of Radiology, The Second Affiliated Hospital of Zhejiang Chinese Medical University, 318 Chao-Wang Road, Hangzhou, 310005 Zhejiang China

**Keywords:** Appendiceal mucinous neoplasms, Radiomics, Appendicitis, Computed tomography, Appendix, Mucocele

## Abstract

**Objective:**

To develop and validate a radiomics nomogram based on computed tomography (CT) to distinguish appendiceal mucinous neoplasms (AMNs) from appendicitis with intraluminal fluid (AWIF).

**Method:**

A total of 211 patients from two medical institutions were retrospectively analysed, of which 109 were pathologically confirmed as having appendicitis with concomitant CT signs of intraluminal fluid and 102 as having AMN. All patients were randomly assigned to a training (147 patients) or validation cohort (64 patients) at a 7:3 ratio. Radiomics features of the cystic fluid area of the appendiceal lesions were extracted from nonenhanced CT images using 3D Slicer software. Minimum redundancy maximum relevance and least absolute shrinkage and selection operator regression methods were employed to screen the radiomics features and develop a radiomics model. Combined radiomics nomogram and clinical-CT models were further developed based on the corresponding features selected after multivariate analysis. Lastly, receiver operating characteristic curves, and decision curve analysis (DCA) were used to assess the models’ performances in the training and validation cohorts.

**Results:**

A total of 851 radiomics features were acquired from the nonenhanced CT images. Subsequently, a radiomics model consisting of eight selected features was developed. The combined radiomics nomogram model comprised rad-score, age, and mural calcification, while the clinical-CT model contained age and mural calcification. The combined model achieved area under the curves (AUCs) of 0.945 (95% confidence interval [CI]: 0.895, 0.976) and 0.933 (95% CI: 0.841, 0.980) in the training and validation cohorts, respectively, which were larger than those obtained by the radiomics (training cohort: AUC, 0.915 [95% CI: 0.865, 0.964]; validation cohort: AUC, 0.912 [95% CI: 0.843, 0.981]) and clinical-CT models (training cohort: AUC, 0.884 [95% CI: 0.820, 0.931]; validation cohort: AUC, 0.767 [95% CI: 0.644, 0.863]). Finally, DCA showed that the clinical utility of the combined model was superior to that of the clinical CT and radiomics models.

**Conclusion:**

Our combined radiomics nomogram model constituting radiomics, clinical, and CT features exhibited good performance for differentiating AMN from AWIF, indicating its potential application in clinical decision-making.

## Introduction

Appendiceal mucinous neoplasms (AMNs) are rare cystic tumours accounting for < 1% of all appendectomies. The microscopic pathology of AMNs is characterised by mucous epithelial hyperplasia and extracellular mucus production, grossly visible as mucus accumulation in the appendiceal lumen (Yu et al. 2020; Ahadi et al. 2021; Shaib et al. 2017). In contrast, appendicitis is one of the most common acute abdominal conditions, with intraluminal fluid as a computed tomography (CT) feature (Mahankali et al. 2021; Moteki et al. 2010). Therefore, distinguishing AMN from appendicitis can occasionally be challenging owing to the common manifestation of intraluminal fluid, leading to their frequent preoperative misdiagnosis in the clinic (Basak et al. 2018; Soon et al. 2020; To et al. 2014; Yilmaz et al. 2013). Furthermore, AMN and appendicitis require distinct treatments (Di Saverio et al. 2020; Glasgow et al. 2019; Moris et al. 2021). In the case of AMN, surgical resection is necessitated. Intraoperatively, the integrity of the appendix wall and the surrounding peritoneum should be evaluated, and iatrogenic damage to the lesion should be prevented to avoid the spread of mucous into the abdominal cavity (Glasgow et al. 2019). With regard to appendicitis treatment, this condition can be managed using conservative anti-inflammatory therapy or surgical removal. Surgery can be performed by conventional excision, wherein the evaluation of the appendix wall and surrounding peritoneum conditions is unnecessary (Di Saverio et al. 2020; Moris et al. 2021). Thus, accurately differentiating AMN from appendicitis prior to surgery is extremely crucial.

Currently, various imaging modalities are vital for diagnosing appendiceal diseases. The short-axis diameter of the appendix is considered helpful in differentiating AMN from acute appendicitis (Bennett et al. 2009; Saylam et al. 2013). However, small-sized or early-growing AMNs are difficult to diagnose with conventional imaging. Additionally, diffusion-weighted imaging and apparent diffusion coefficient sequence of magnetic resonance imaging (MRI) have been suggested to help detect AMN and appendicitis by determining the properties of the luminal fluid. However, considering that this approach has been described in only one case report, validation by studies using large samples is warranted (INOUE et al. 2020). Therefore, a new diagnosis method is required to more accurately differentiate AMN from appendicitis.

Radiomics analysis, a recently developed technique to extract extensive quantitative features from medical images, has been applied to support clinical decision-making by improving prediction, prognosis, and diagnostic accuracy (Sah et al. 2019; Ma et al. 2022). However, to our knowledge, relevant research investigating the implementation of radiomics analysis to identify appendiceal diseases has not been published.

In this study, we initially classified appendicitis with intraluminal fluid (AWIF) into a separate category to distinguish it from AMN, consistent with the disease identification process in a real-world clinical setting. Furthermore, we aimed to develop and validate a combined nomogram incorporating radiomics, clinical, and CT features to differentiate AMN from AWIF.

## Materials and methods

### Patients

**Fig. 1:**
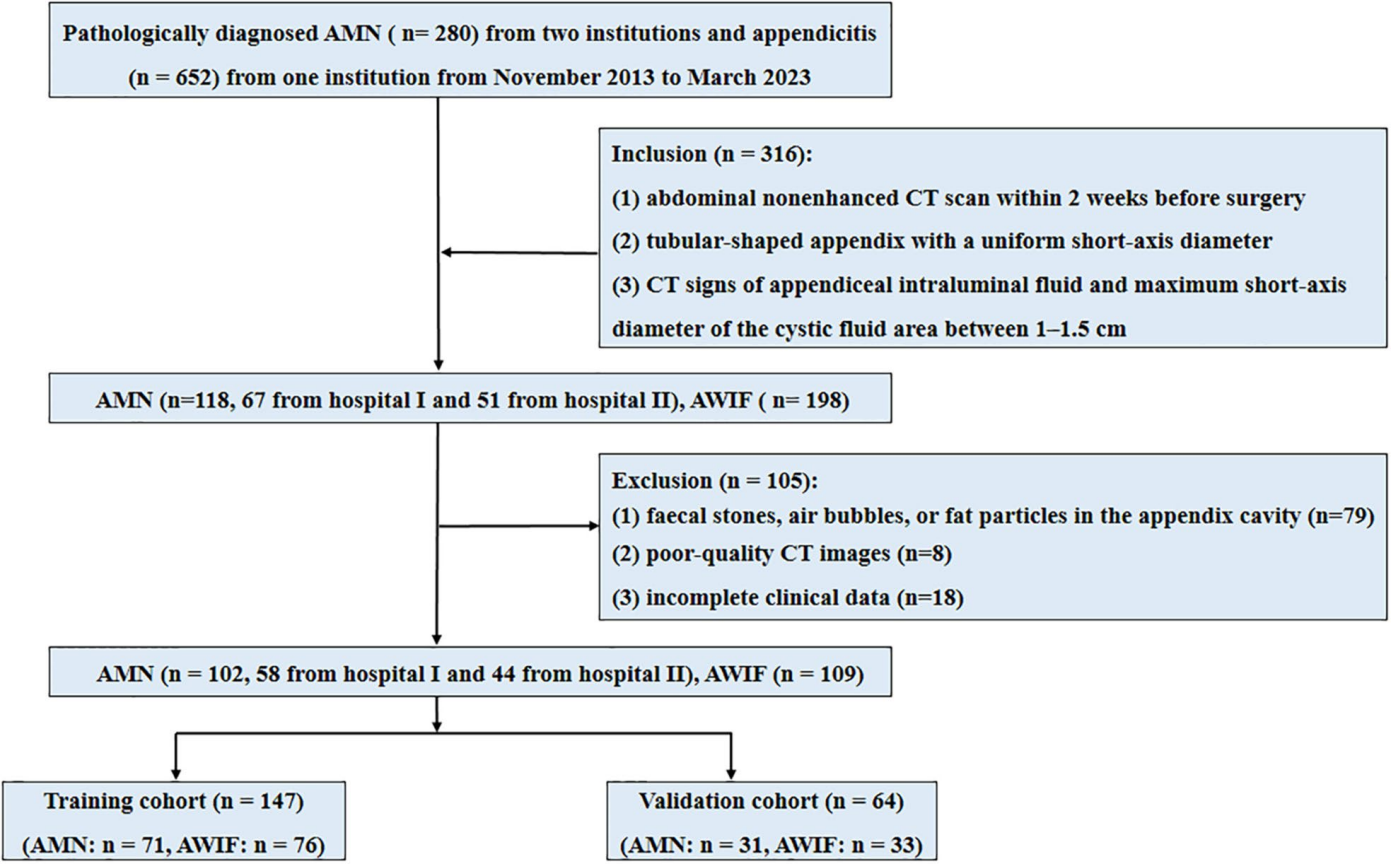
Flowchart of patient selection

The institutional review boards of the two involved medical centres approved this study and waived the requirement for informed consent due to its retrospective design. From November 2013 to March 2023, 102 patients with AMN (58 from hospital I and 44 from hospital II) and 109 patients with appendicitis from hospital II were enrolled. The patient inclusion criteria were as follows: (1) histopathological diagnosis of AMN or appendicitis; (2) abdominal nonenhanced CT scan within 2 weeks before surgery; (3) tubular-shaped appendix with a uniform short-axis diameter; and (4) CT signs of appendiceal intraluminal fluid and maximum short-axis diameter of the cystic fluid area between 1–1.5 cm. The patient exclusion criteria were as follows: (1) faecal stones, air bubbles, or fat particles in the appendix cavity; (2) poor-quality CT images; or (3) incomplete clinical data. A total of 211 patients were randomly assigned to a training (*n* = 147) or validation cohort (*n* = 64) (Fig. 1).

Additionally, patient clinical data, including sex, age, right lower abdominal pain, fever, and white blood cell increase (> 9.5 × 10^9^), were recorded.

### Image acquisition and analysis

Two 64-slice and one 16-slice spiral CT scanners (Siemens Healthineers 64-slice, Forchheim, Germany; or Philips Medical Systems 64 and 16-slice, Cleveland, OH, USA) were used for abdominal nonenhanced CT examinations. The scanning parameters were as follows: tube voltage, 120 kVp; tube current, 220 mA; field of view, 350 × 350 mm; pitch 0.8 and 1.2; slice thickness, 2 mm; and slice interval, 2 mm. Patients were imaged in a supine position, and the scan range is from 2 cm above the xiphoid process to the lower border of the pubic symphysis.

CT signs, including periappendiceal fat stranding, mural calcification, periappendiceal free fluid, luminal fluid attenuation, short-axis diameter of the luminal fluid area, thin line mural, and mural thickening of the cecum, were analysed by two abdominal radiologists who had 12 and 32 years of experience and were blinded to the clinical and pathological details. The conclusions of the 32-year-old abdominal radiologist were used in cases of data discrepancies.

### Image segmentation and features extraction

**Fig. 2:**
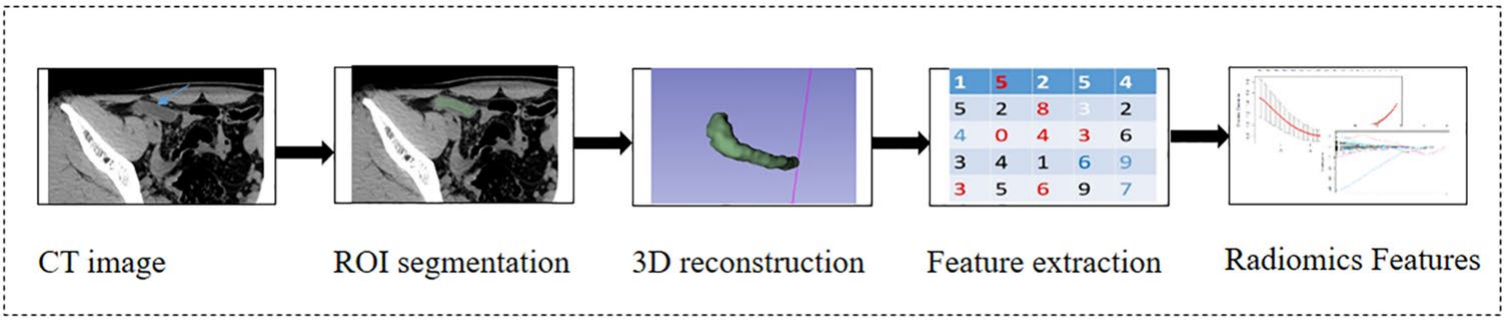
Radiomics workflow

Nonenhanced CT images of the patients with AMN or AWIF were used for radiomics analysis via 3D Slicer software (version 5.0.3; https://www.slicer.org). In each layer of the images, the regions of interest (ROIs) were drawn carefully along the boundary of the cystic fluid area by one abdominal radiologist with 13 years of experience, after 30 of the 211 patients were randomly selected by the second radiologist with three years of experience for drawing the ROIs. The two radiologists were blinded to the clinical data and diagnosis. A total of 851 radiomics features were extracted from each case of AMN or AWIF using the radiomics package of the 3D Slicer software. These features were then categorised into the following eight groups: (a) original-first-order features, (b) original-shape features, (c) original-glcm features, (d) original-gldm features, (e) original-glrlm features, (f) original-glszm features, (g) original-ngtdm features, and (h) wavelet features. The radiomics workflow is shown in Fig. 2.

### Radiomics features selection

Minimum redundancy maximum relevance (mRMR) and least absolute shrinkage and selection operator (LASSO) regression methods were performed to screen the radiomics features. First, mRMR was used to reduce the redundant and irrelevant features, resulting in 50 radiomics features. Next, we employed LASSO regression with tenfold cross-validation to display the model coefficients of each feature at lambda one standard error (lambda.1se). Based on the number of features indicated by the vertical line on the right side of the cross-validation plot, we conducted feature selection based on the magnitude of the coefficients' absolute values. Further, intraclass correlation coefficients (ICCs) were determined for the selected features extracted by the two radiologists. The conclusions of the 13-year-old abdominal radiologist were used in cases of the selected data discrepancies. Finally, the Spearman rank correlation test was applied to assess the association among the selected features.

### Radiomics model development and evaluation

The rad-score of each patient was calculated by summing the LASSO coefficient-based weights of the selected features, which were utilized as potential predictors in constructing the radiomics model. The Wilcoxon test was then used to compare the difference between the rad scores of AMN and AWIF. Additionally, the discrimination power of the radiomics model was evaluated using the area under the receiver operating characteristic (ROC) curve (AUC) in the training and validation cohorts based on the rad-scores.

### Radiomics nomogram construction and evaluation

After univariate analysis, the independent predictors of AMN were selected by multivariate analysis. Using these independent predictors, a combined radiomics nomogram that integrated radiomics, clinical, and CT features was established. A clinical-CT model was also established based on the clinical and CT features selected after multivariate analysis. Furthermore, the goodness of fit of the combined radiomics nomogram model was assessed using the Hosmer–Lemeshow test. The performance of the combined radiomics nomogram and clinical-CT models was also evaluated in the training and validation cohorts by plotting their ROC curves and calculating their AUC values. Lastly, the clinical utility of the models was examined by plotting their DCA curves.

### Statistical analysis

Categorical variables were analysed via the chi-squared or Fisher’s exact tests, whereas continuous variables were compared using the independent t or Wilcoxon tests. Interobserver variability was assessed using the ICC model. Lastly, ROC curves, Hosmer–Lemeshow test, and DCA were used to determine the reliability and usefulness of the models. SPSS 23.0 (IBM, Armonk, NY, USA), R (v.4.1.2, Vienna, Austria), and MedCalc v. 19.0 (MedCalc Software, Ostend, Belgium) software were used to perform the statistical analyses. A *p* value of < 0.05 was considered statistically significant.

## Results

### Clinical and CT features

The ICCs of the seven CT features were all ≥ 0.75. The clinical and CT features of the patients with AMN or AWIF are depicted in Table 1. Age, periappendiceal fat stranding, and mural calcification exhibited significant differences between the AMN and AWIF groups in the training and validation cohorts (*p* < 0.05), whereas white blood cell increase was significantly different only in the training cohort (*p* < 0.05). The remaining clinical and CT features demonstrated no significant differences between the two groups (*p* > 0.05).

**Table 1:** Comparison clinical and CT features between AMN and AWIF

Characteristic	Training cohort			Validation cohort		
	AMN (*n* = 71)	AWIF (*n* = 76)	P value	AMN (*n* = 31)	AWIF (*n* = 33)	P value
Sex [*n* (%)]			0.824^#^			0.846^#^
Male	37 (51.11)	41 (53.95)		12 (38.71)	12 (36.36)	
Female	34 (47.89)	35 (46.05)		19 (61.29)	21 (63.64)	
Age (year)	63.72 ± 13.61	44.17 ± 16.67	**0.005**^**&**^	58.71 ± 15.34	46.76 ± 20.88	**0.004**^**&**^
Right lower abdominal pain [*n* (%)]	39 (54.93)	50 (65.79)	0.178^#^	14 (45.16)	21 (63.64)	0.138^#^
Fever [*n* (%)]	6 (8.45)	11 (14.47)	0.254^#^	2 (6.45)	5 (15.15)	0.265^#^
White blood cell increase [*n* (%)]	26 (36.62)	43 (56.58)	**0.015**^**#**^	13 (41.94)	19 (57.58)	0.211^#^
Periappendiceal fat stranding [*n* (%)]	21 (29.58)	45 (59.21)	** < 0.001**^**#**^	8 (25.81)	18 (54.55)	**0.019**^#^
Mural calcification [*n* (%)]	38 (53.52)	5 (6.58)	** < 0.001**^#^	12 (38.71)	2 (6.06)	**0.002**^#^
Periappendiceal free fluid [*n* (%)]	7 (9.86)	2 (2.63)	0.068^#^	3 (9.68)	3 (9.09)	0.936^#^
Luminal fluid attenuation (Hu)	12.38 ± 4.80	11.87 ± 5.26	0.155^&^	11.56 ± 4.01	11.91 ± 5.31	0.053^&^
Short-axis diameter of the luminal fluid area [*n* (%)]	12.68 ± 1.34	12.60 ± 1.80	0.757^&^	12.84 ± 1.42	12.64 ± 1.49	0.573^&^
Thin line mural [*n* (%)]	52 (73.24)	56 (73.68)	0.951^#^	25 (80.65)	20 (60.61)	0.079^#^
Mural thickening of the cecum [*n* (%)]	3 (4.23)	5 (6.58)	0.530^#^	0 (0)	1 (3.03)	1.000*

*p* value < 0.05 written in bold indicates a significant difference

*AMN* appendiceal mucinous neoplasms, *AWIF* appendicitis with intraluminal fluid

^&^t-test; ^#^Chi-square test; *Fisher's exact tests

### Radiomics features selection and radiomics model building

**Fig. 3:**
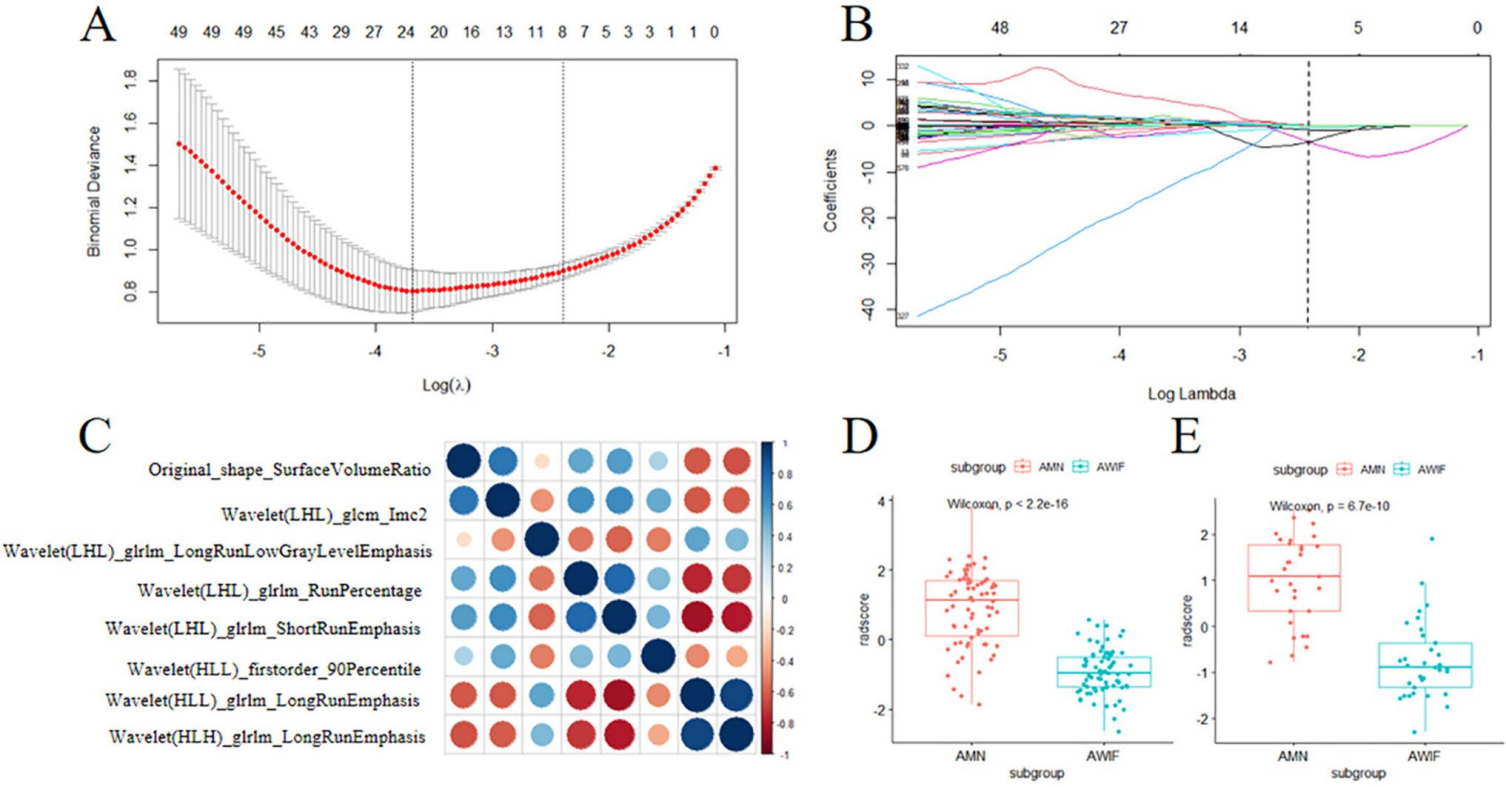
Radiomics feature selection and rad-score display. **A** A tuning parameter (*λ*) of the LASSO model by tenfold cross-validation was selected based on minimum criteria. The *y*-axis displays binomial deviance. The upper *x*-axis shows the number of radiomics features, and the lower *x*-axis shows the log(*λ*) value. In this study, the dotted line on the right was chosen, which represented an optimal *λ* value, with log(*λ*) = − 2.4 and eight features selected. **B** LASSO coefficient profiles of the radiomics features. Each curve represents the coefficient change for every feature. The vertical line showed that the optimal lambda value produced eight nonzero coefficients. **C** Spearman rank correlation among eight features selected by LASSO regression in the training cohort. **D**, **E**. The rad-scores of the AMN and AWIF patients in the training (**D**) and validation cohorts (**E**)

Eight features were eventually screened using LASSO regression (Fig. 3A, B), including one original shape feature and seven wavelet features. The Spearman rank correlation test results are illustrated in Fig. 3C, indicating that the collinearity among the eight selected features was low. The ICCs of the eight selected features were all ≥ 0.75, suggesting good agreement between the two radiologists.

The rad scores were calculated based on the following formula:

$$ \begin{gathered} {\text{Rad - score}}\, = \,{5}.{6925}\, + \,\left( { - 0.{4}0{3}0} \right)*{\text{Original}}\_{\text{shape}}\_{\text{SurfaceVolumeRatio}}\, \hfill \\ \quad \quad + \,\left( { - 0.{9231}} \right)*{\text{ Wavelet}}\left( {{\text{LHL}}} \right)\_{\text{glcm}}\_{\text{Imc2}}\, + \,0.0{1}0{5}*{\text{ Wavelet}}\left( {{\text{LHL}}} \right)\_{\text{glrlm}}\_{\text{LongRunLowGrayLevelEmphasis}}\, \hfill \\ \quad \quad + \,\left( { - {3}.{7752}} \right)*{\text{Wavelet}}\left( {{\text{LHL}}} \right)\_{\text{glrlm}}\_{\text{RunPercentage}}\, + \,\left( { - {3}.{3}0{77}} \right)*{\text{Wavelet}}\left( {{\text{LHL}}} \right)\_{\text{glrlm}}\_{\text{ShortRunEmphasis}} \hfill \\ \quad \quad \, + \,\left( { - 0.0{334}} \right)*{\text{ Wavelet}}\left( {{\text{HLL}}} \right)\_{\text{firstorder}}\_{9}0{\text{Percentile}}\, + \,0.0{928}*{\text{Wavelet}}\left( {{\text{HLL}}} \right)\_{\text{glrlm}}\_{\text{LongRunEmphasis}}\, \hfill \\ \quad \quad + \,0.{1}0{35}*{\text{ Wavelet}}\left( {{\text{HLH}}} \right)\_{\text{glrlm}}\_{\text{LongRunEmphasis}}. \hfill \\ \end{gathered} $$

Furthermore, the rad-scores of AMN and AWIF were compared between the training and validation cohorts, demonstrating a significant difference (P < 0.05; Fig. 3D, E). Finally, the rad scores were used as predictors in constructing the radiomics model.

### Development of the combined radiomics nomogram

**Fig. 4:**
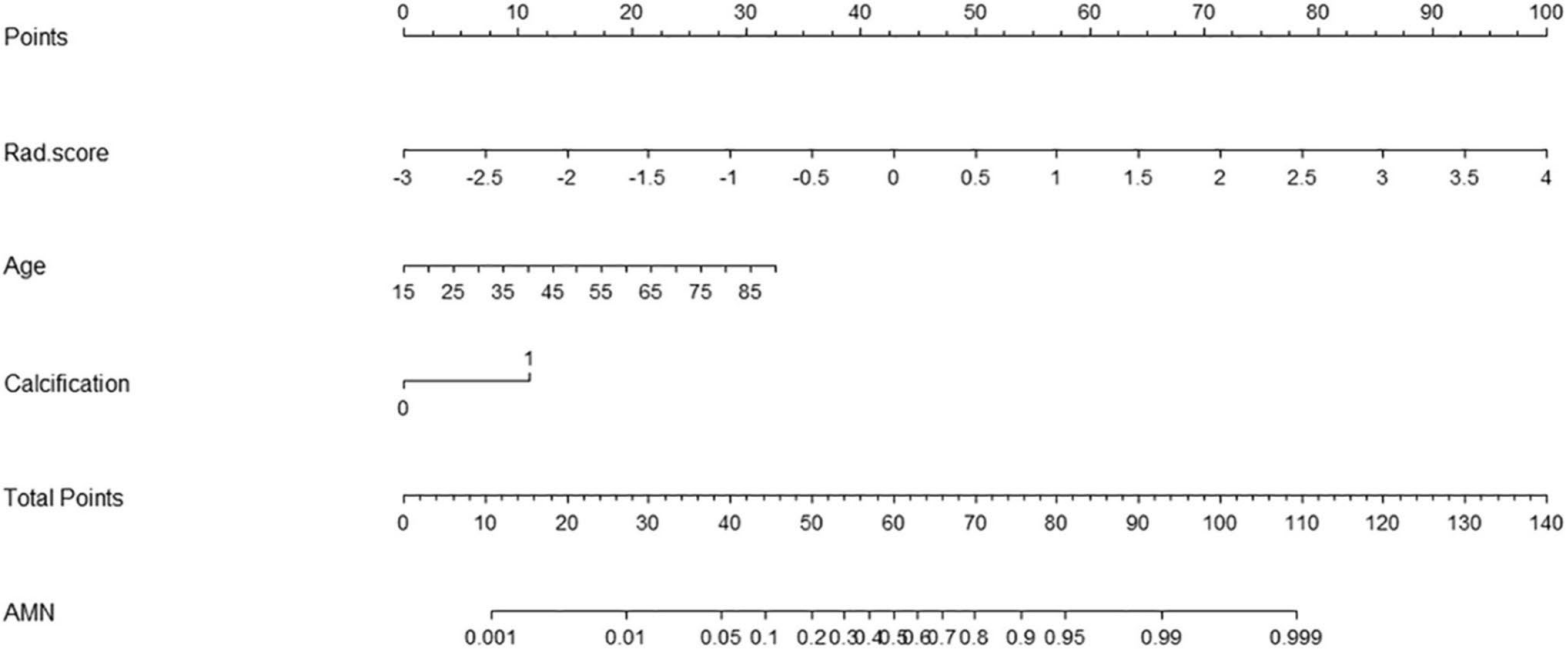
Nomogram incorporating radiomics, clinical, and CT features for differentiating AMN from AWIF

Multivariate analysis showed that age, mural calcification, and rad-score were independent predictors to differentiate AMN from AWIF (Table 2). The variance inflation factors of the independent predictors were all < 2, suggesting no collinearity among them. Subsequently, a combined model was developed by incorporating rad-score, age, and mural calcification and presented as a nomogram (Fig. 4). The Hosmer–Lemeshow test also revealed *p*-values of 0.401 and 0.199 for the training and validation cohorts, respectively, indicating no deviation from the goodness of fit of the combined model. Additionally, a clinical-CT model was constructed by incorporating age and mural calcification.

**Table 2:** Univariate and multivariate analysis of features for differientiaing AMN from AWIF

Variable	OR	Univariate analysis	*P*	OR	Multivariate analysis	*P*	VIF
		95%CI			95%CI		
Sex	0.929	0.486–1.776	0.824				
Age	1.081	1.053–1.109	** < 0.001**	1.062	1.026–1.101	**0.001**	1.230
Right lower abdominal pain	0.634	0.326–1.233	0.179				
Fever	0.545	0.190–1.563	0.259				
White blood cell increase	0.443	0.229–0.860	**0.016**	0.594	0.177–1.998	0.400	1.075
Periappendiceal fat stranding	0.289	0.146–0.574	** < 0.001**	0.447	0.142–1.405	0.168	1.187
Mural calcification	16.352	5.898–45.335	** < 0.001**	4.661	1.147–18.943	**0.031**	1.555
Periappendiceal free fluid	4.047	0.812–20.179	0.088				
Luminal fluid attenuation	1.020	0.956–1.089	0.542				
Short-axis diameter of the luminal fluid area	1.033	0.842–1.268	0.757				
Thin line mural	0.977	0.470–2.034	0.951				
Mural thickening of the cecum	0.626	0.144–2.723	0.533				
Rad-score	8.774	4.416–17.431	** < 0.001**	7.393	3.281–16.658	** < 0.001**	1.713

*p* value < 0.05 written in bold indicates a significant difference

*AMN* appendiceal mucinous neoplasms, *AWIF* appendicitis with intraluminal fluid, *CI* confidence interval, *OR* odds ratio, *VIF* variance inflation factor

### Performance and clinical utility of the models

**Fig. 5:**
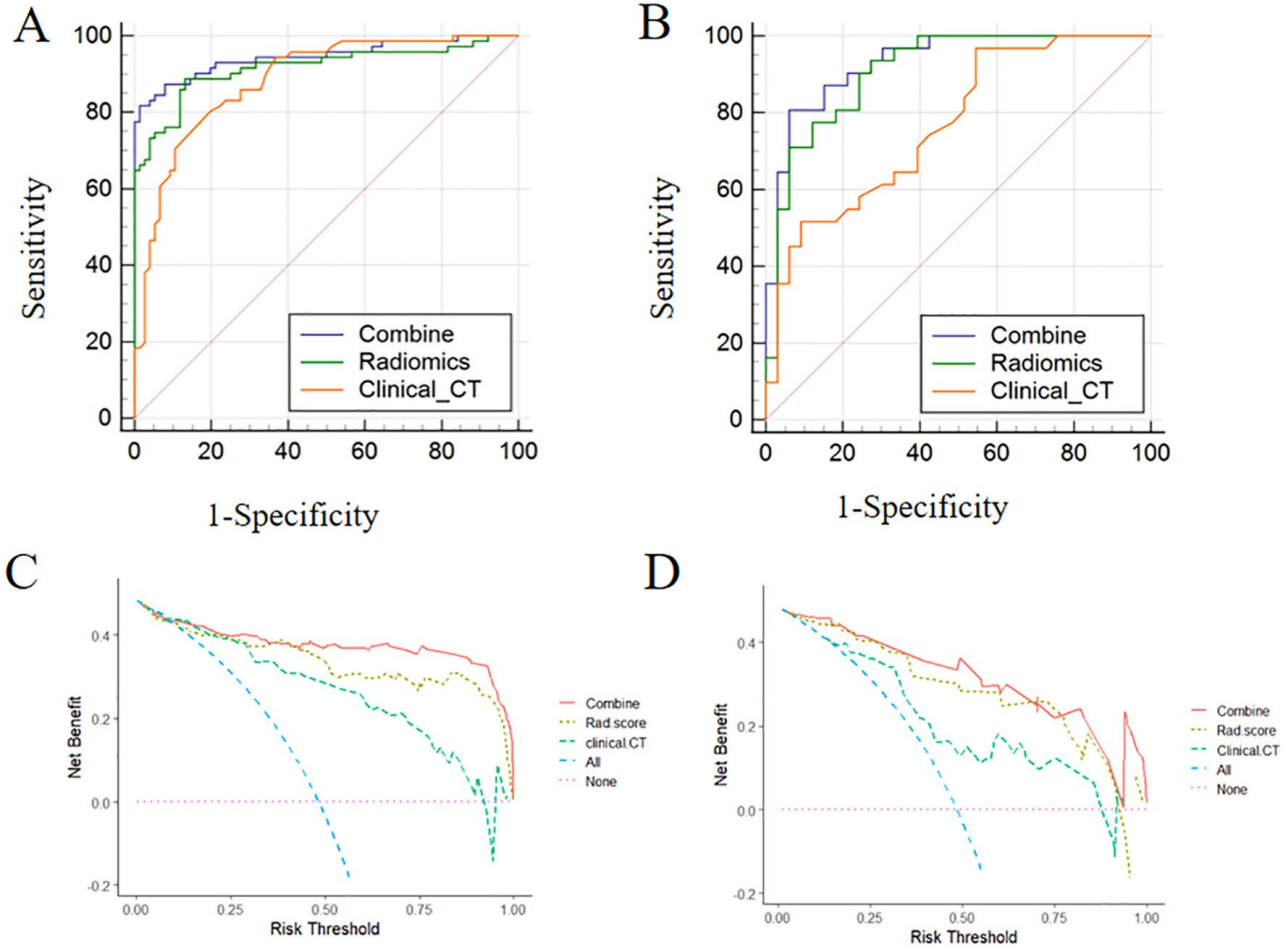
Performance and clinical utility of the models. ROC curves of the combined, radiomics, and clinical-CT models for differentiating AMN from AWIF in the training (**A**) and validation cohorts (**B**). DCA for the combined, radiomics, and clinical-CT models in the training (**C**) and validation cohorts (**D**)

ROC curves of the combined, radiomics, and clinical-CT models are displayed in Fig. 5A, B and the detailed performance results of the three models are listed in Table 3. In the training cohort, the combined model provided the greatest discriminatory power between the AMN and AWIF groups, exhibiting an AUC of 0.945 (95% confidence interval [CI]: 0.895, 0.976) that was larger than the radiomics (AUC, 0.915 [95% CI: 0.865, 0.964]) and clinical-CT models (AUC, 0.884 [95% CI: 0.820, 0.931]). Additional examination using the DeLong test revealed differences between the combined and the clinical-CT models in the training cohort (combined vs clinical-CT, *p* = 0.008). However, the radiomics model did not differ from the other two models (radiomics vs combined, *p* = 0.053; radiomics vs clinical-CT, *p* = 0.331). Similarly, in the validation cohort, the combined model achieved the highest AUC of 0.933 (95% CI: 0.841, 0.980), demonstrating better predictive power than the radiomics (AUC, 0.912 [95% CI: 0.843, 0.981]) and clinical-CT models (AUC, 0.767 [95% CI: 0.644, 0.863]). Additionally, the clinical-CT model differed from the combined and radiomics models in the validation cohort (clinical-CT vs combined, *p* = 0.007; clinical-CT vs radiomics, *p* = 0.04), while no differences were detected between the combined and radiomics models (combined vs radiomics, *p* = 0.163).

**Table 3:** Predictive performance of clinical-CT model, radiomics model and combined model

Model	Training cohort			Validation cohort		
	AUC (95%CI)	Specificity	Sensitivity	AUC (95%CI)	Specificity	Sensitivity
Combined model	0.945 (0.895–0.976)	0.987	0.817	0.933 (0.841–0.980)	0.939	0.806
Radiomics model	0.915 (0.865–0.964)	0.868	0.873	0.912 (0.843–0.981)	0.727	0.935
Clinical-CT model	0.884 (0.820–0.931)	0.816	0.789	0.767 (0.644–0.863)	0.909	0.516

*CI* confidence internal, *AUC* area under curve

Additionally, DCA demonstrated that the curve of the combined model (red curve) was higher than that of the radiomics (yellow curve) and clinical-CT models (green curve) as well as the ‘all’ (blue curve) and ‘none’ (pink curve) scenarios in the training and validation cohorts over a wide range of threshold probabilities (Fig. 5C, D). These findings suggested good clinical utility of the combined model for differentiating AMN from AWIF.

**Fig. 6:**
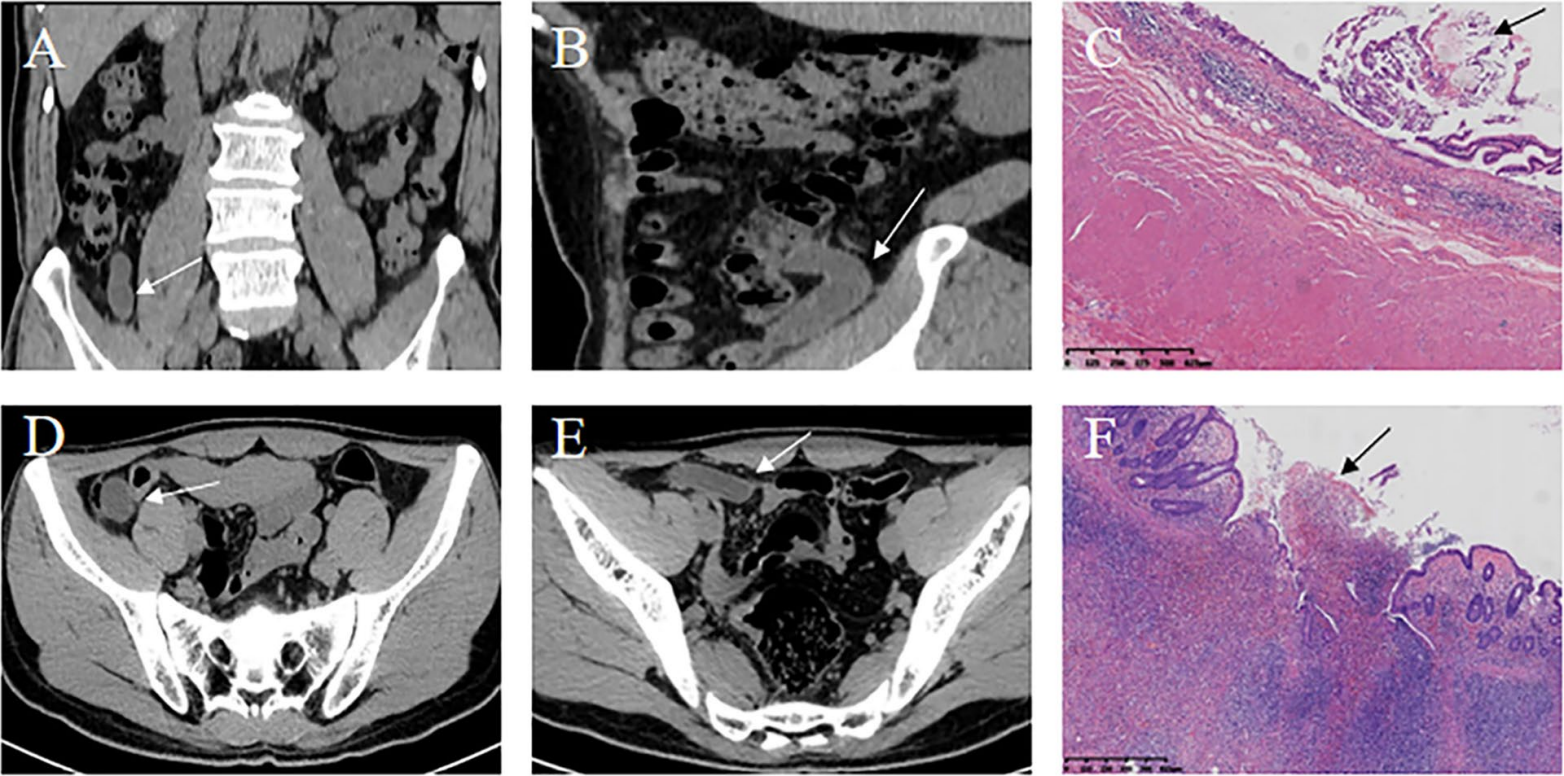
The combined radiomics nomogram model for differentiating AMN from AWIF. **A**–**C** Patient 1: A 64-year-old man presented with recurrent right lower abdominal pain for 6 months. **A** Coronal and **B** sagittal CT showed a tubular cystic lesion (white arrow) in the ileocaecal region. **C**. Postoperative pathology (haematoxylin–eosin staining; magnification, × 4) demonstrated residual mucin (black arrow) in the appendix cavity, mucosal surface atrophy, disappearance of submucosal lymphatic tissue, and AMN diagnosis. The rad-score of patient 1 was (− 0.089). In the nomogram for patient 1, age scored 21 points, and the rad-score was given 42 points, yielding a total score of 63. According to the nomogram, the probability of AMN for patient 1 was about 60%, consistent with the pathological result. **D**–**F** Patient 2: A 28-year-old man presented with mid-upper abdominal pain for 5 h. **D**, **E** Axial CT revealed a tubular cystic lesion in the right lower abdomen. **F** Postoperative pathology (haematoxylin–eosin staining; magnification, × 4) indicated that the whole layer of the appendix wall was infiltrated by neutrophils, with a few of the inflammatory cells entering the tube cavity (black arrow). A diagnosis of suppurative appendicitis was considered. The rad-score of patient 2 was (− 0.54). In the nomogram for patient 2, age received 6 points, while the rad-score scored 35, reaching a total score of 41. Based on the nomogram, the probability of AMN in patient 2 was ≤ 10%, indicating the diagnosis of another disease (AWIF) corresponding to the pathological result

Lastly, the combined radiomics nomogram was applied in practical cases, and its predictive efficiency was confirmed (Fig. 6).

## Discussion

The preoperative differentiation of AMN from AWIF is crucial for surgeons to plan appropriate therapy strategies. In the current study, we developed and validated a combined radiomics nomogram model that incorporated rad-score, age, and mural calcification for distinguishing AMN from AWIF. The combined model showed great discrimination, achieving AUCs of 0.945 and 0.933 in the training and validation cohorts, respectively, which were larger than those of the radiomics (training cohort: AUC, 0.915; validation cohort: AUC, 0.912) and clinical-CT models (training cohort: AUC, 0.884; validation cohort: AUC, 0.767). Further, the Hosmer–Lemeshow test demonstrated that the combined model was a good fit in the training (*p* = 0.401) and validation cohorts (*p* = 0.199). DCA also confirmed that the combined model had clinical utility superior to the radiomics and clinical-CT models.

The cystic fluid in AMN is histologically composed of mucin, whereas the intraluminal fluid in appendicitis consists of purulent material (INOUE et al. 2020; Ahadi et al. 2021; Moris et al. 2021). Thus, identifying the nature of these two fluids via CT or ultrasound can be difficult. In contrast, radiomics is an advanced method involving high-throughput extraction of features from medical images and subsequently analysing the numerous quantitative imaging characteristics representing underlying histologic features that could not be visually observed (Calabrese et al. 2021; Dong et al. 2022). Several studies have reported using radiomic methods to differentiate between cystic lesions in other organs. For example, Fang et al. found that MRI T2-weighted imaging-based radiomics showed good performance in discriminating between pancreatic serous and mucinous cystic neoplasms (training cohort: AUC, 0.93; validation cohort: AUC, 0.86) (Fang et al. 2022). Another radiomics model based on CT developed by Pan et al. also exhibited great potential for discriminating between ovarian serous and mucinous cystadenoma (Pan et al. 2020). Those two studies were based on different histological characteristics of the cystic fluid area, providing a research direction for our study. In the current study, five of the eight radiomics features selected via LASSO regression quantitatively reflected the homogeneity and complexity of the focal parameters of the grey-level run-length matrix (GLRLM) (Chen et al. 2022). Therefore, radiomics can be used to identify the different pathological features of the cystic fluid in AMN and AWIF.

Previous studies have explored applying clinical and conventional imaging data to differentiate between AMN and appendicitis. For instance, the short-axis diameter of the appendix is considered to provide a high discriminatory value (Lien et al. 2006; Saylam et al. 2013). Specifically, a short-axis diameter of > 15 mm was less predictive of appendicitis. Additionally, Saylam et al. concluded that a short-axis diameter of < 10 mm had lower predictability for AMN. Therefore, we limited the maximum short-axis diameter of the cystic fluid area in the appendiceal lumen to 1–1.5 cm and excluded lesions with highly specific signs for appendicitis diagnosis, such as faecal stones and air bubble signs in the lumen, consistant with the differential diagnosis scenarios in real clinical practice. Saylam et al. also revealed that the mean age of patients with appendiceal mucinous tumours was 56.57 years, whereas that of patients with appendicitis was 48.91 years (Saylam et al. 2013). This observation was close to our findings, suggesting that age may be a useful indicator to differentiate AMN from AWIF. Moreover, our multivariate analysis demonstrated that mural calcification might help differentiate AMN from AWIF. This result was in line with many previous studies that suggested that mural calcification is a typical AMN feature (Brassil et al. 2022; Monsonis et al. 2021). The mechanism of mural calcification may be a pushing growth pattern of AMN that leads to fibrosis with dystrophic mural calcification of the appendix (Ahadi et al. 2021). In summary, according to the prior research findings and our multivariate analysis, age (odds ratio [OR]: 1.062) and mural calcification (OR: 4.661) may be effective predictors in the model construction.

Accordingly, we developed and validated a combined nomogram model to differentiate AMN from AWIF by incorporating rad-score, age, and mural calcification. To the best of our knowledge, similar models discriminating AMN from AWIF have not been reported, highlighting the novelty of our study. Furthermore, our combined model achieved AUCs of 0.945 and 0.933 in the training and validation cohorts, respectively. These values were higher than those obtained by the radiomics and clinical-CT models, indicating that the combined model had the best predictive power. Moreover, the DeLong test demonstrated differences between the combined and clinical-CT models in the training and validation cohorts. These results suggested that the radiomics features based on the cystic fluid area could improve the ability to discriminate AMN from AWIF. However, the clinical-CT model we developed only had an AUC of 0.767 and a sensitivity of 0.516 in the validation cohort, both of which were lower than the results reported by Saylam et al. (AUC: 0.855; sensitivity: 0.765) (Saylam et al. 2013). We speculate that the reason for this discrepancy may be attributed to the biased results resulting from the small sample size of the validation cohort or to the diagnostic difficulties introduced in the clinical-CT model due to our restrictions on specific features, including the maximum short-axis diameter, faecal stones, and air bubbles. Moreover, nomograms are an intuitive and quantifiable tool widely used in previous studies (Wang et al. 2022; Zheng et al. 2021). We visualised our combined model as a nomogram, making it convenient for clinical application.

Nevertheless, our study has several limitations that should be considered. First, the study had a small sample size and lacked external validation. To address this issue, we hope to expand the sample size further and include external datasets for validation in the future. Second, nonenhanced CT images were used instead of enhanced CT images due to the unavailability of enhanced CT data. Thus, the features of the appendiceal mural enhancement could not be assessed. We hope to investigate AMN and AWIF further using enhanced CT images when the data volume is sufficient. Finally, the manual segmentation of the cystic fluid area conducted by the study radiologists was time-consuming. Consequently, we aim to develop an automatic segmentation method in the future.

In conclusion, our combined radiomics nomogram constituting rad-score, age, and mural calcification is a potentially valuable tool for differentiating AMN from AWIF.

## Data Availability

The datasets and code used and/or analyzed during the current study are available from the corresponding author upon reasonable request.
